# Building local capacity for genomics research in Africa: recommendations from analysis of publications in Sub-Saharan Africa from 2004 to 2013

**DOI:** 10.3402/gha.v9.31026

**Published:** 2016-05-12

**Authors:** Babatunde O. Adedokun, Christopher O. Olopade, Olufunmilayo I. Olopade

**Affiliations:** 1 Department of Epidemiology and Medical Statistics, College of Medicine, University of Ibadan, Ibadan, Nigeria; 2 Centre for Global Health, Department of Medicine, University of Chicago, Chicago, IL, USA; 3 Center for Clinical Cancer Genetics and Global Health, Department of Medicine, University of Chicago, Chicago, IL, USA

**Keywords:** capacity building, genomics, health research, sub-Saharan Africa, bibliometric analysis

## Abstract

**Background:**

The poor genomics research capacity of Sub-Saharan Africa (SSA) could prevent maximal benefits from the applications of genomics in the practice of medicine and research. The objective of this study is to examine the author affiliations of genomic epidemiology publications in order to make recommendations for building local genomics research capacity in SSA.

**Design:**

SSA genomic epidemiology articles published between 2004 and 2013 were extracted from the Human Genome Epidemiology (HuGE) database. Data on authorship details, country of population studied, and phenotype or disease were extracted. Factors associated with the first author, who has an SSA institution affiliation (AIAFA), were determined using a Chi-square test and multiple logistic regression analysis.

**Results:**

The most commonly studied population was South Africa, accounting for 31.1%, followed by Ghana (10.6%) and Kenya (7.5%). About one-tenth of the papers were related to non-communicable diseases (NCDs) such as cancer (6.1%) and cardiovascular diseases (CVDs) (4.3%). Fewer than half of the first authors (46.9%) were affiliated with an African institution. Among the 238 articles with an African first author, over three-quarters (79.8%) belonged to a university or medical school, 16.8% were affiliated with a research institute, and 3.4% had affiliations with other institutions.

**Conclusions:**

Significant disparities currently exist among SSA countries in genomics research capacity. South Africa has the highest genomics research output, which is reflected in the investments made in its genomics and biotechnology sector. These findings underscore the need to focus on developing local capacity, especially among those affiliated with SSA universities where there are more opportunities for teaching and research.

## Introduction

Genomic medicine has experienced astronomical growth in recent years (1, 2). The field of genomics holds great promise for health care and medical research, as the identification of the genetic determinants of disease or other phenotypes will bring about significant improvements in diagnosis, prevention, and treatment of several disease conditions (3, 4). Genomics is particularly attractive for Sub-Saharan African (SSA) countries where new technologies and products from genomics research can help mitigate the heavy burden of infectious and chronic non-communicable diseases (NCDs) (5). In addition, such genomics research should be culturally acceptable to SSA populations (6, 7).

In spite of these exciting developments, there are fears that already existing inequalities in health care access will only worsen as technologies and discoveries resulting from genomics research remain affordable only to those who reside in more developed countries (3, 8). Furthermore, genomics research is likely to be biased toward therapeutic and diagnostic applications for conditions affecting populations in wealthy countries with little or no benefit for most people in low- to middle-income countries (3). There is, thus, an urgent need to invest in capacity building and infrastructure development and to encourage investments by SSA governments into genomics research (5, 9–13). Such efforts will lessen dependence on the market-driven research agenda of the developed world for the health needs of low- to middle-income countries (3).

Currently, the state of infrastructure and capacity of SSA scientists is poor (14, 15). In addition, the current investments by governments in SSA remain very low. In most countries in SSA, health research is less than 0.5% of the national health budget (16). Genomics research is a promising area, and deserves more attention from African governments (5). In response to the challenge of poor research funding in Africa, the Human Heredity and Health (H3Africa) initiative, jointly funded by the National Institutes of Health (NIH) and the Wellcome Trust, recently awarded several millions of dollars in grants to investigators on the African continent (17–19).

Although the H3Africa efforts hold great promise for the transformation of genomics research in Africa through capacity building and better research facilities, there is a need to document the state of local or regional genomics research productivity in order to guide the equitable distribution of resources. Presently, few studies have examined the current local capacity of SSA scientists for genomics research. Lack of data on SSA local and regional capacity could jeopardize the efforts of the H3Africa projects and other similar interventions to build local and regional research capacity in SSA. The objective of this review is to examine existing capacity through author affiliations with genome epidemiology publications in order to make recommendations for local and regional genomics research capacity building in SSA.

## Methods

We extracted and analyzed genomic epidemiology publications with SSA study populations over a 10-year period using the HuGE Pub Lit database. SSA, as defined for the purpose of this review, includes Sudan, which is excluded from the United Nations (UN) definition of SSA but included by UN agencies. The articles used were obtained from the Human Genome Epidemiology (HuGE) published literature database (HuGE Pub Lit). The HuGE Pub Lit was launched in 2001 to track publications related to HuGE (20). Articles in the database include studies of human populations that have been published since October 2000 and have English-language abstracts. In addition, the HuGE Pub Lit includes only publications in which genotypes must have been measured at one or more loci while gene discovery articles, such as linkage analysis and gene mapping for high-risk families, were excluded.

**Fig. 1:**
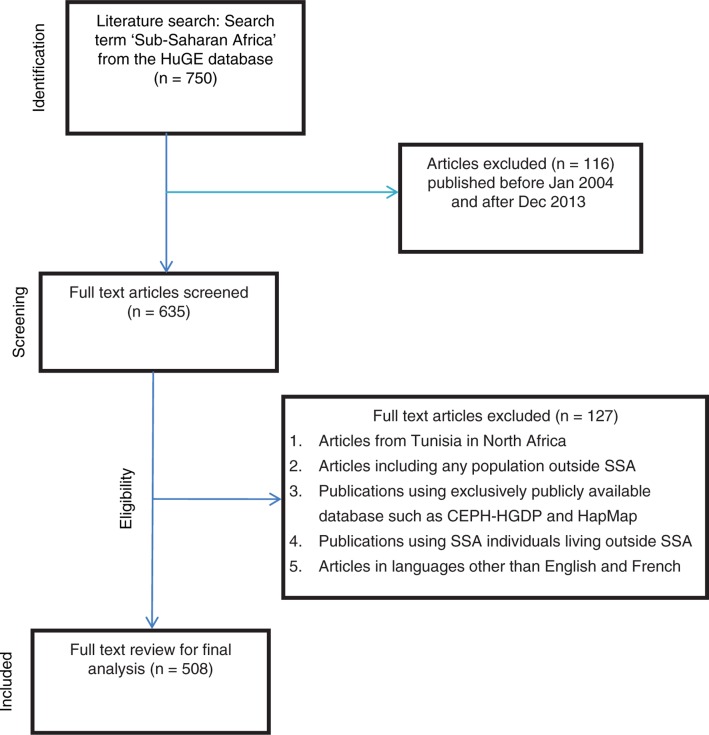
PRISMA (Preferred Items for Systematic Reviews and Meta-analysis) flow diagram for searching and extracting data.

For this analysis, 508 articles published between January 2004 and December 2013 were selected after excluding publications that used data from a foreign population in addition to a SSA population in the same study, and those using data on SSA-born individuals residing in Europe and America (Fig. 1, see Supplementary file for full details). The selection of articles is presented according to PRISMA (Preferred Items for Systematic Reviews and Meta-analysis) guidelines (21). The publications were excluded because research conducted exclusively on African participants would allow a better assessment of local research capacity. One article written in the Russian language was excluded. Studies using data from only SSA populations were chosen in order to adequately assess the contribution of local SSA scientists to genomics research involving local participants. Information extracted from each of the 508 articles includes the year of publication, affiliation of the first author, presence of any African author, and affiliation of the first author from a SSA institution. In cases in which there were multiple affiliations for an author, such an author was still classified as being affiliated with SSA as long as there was a SSA institution among the affiliations. Other variables extracted include the country of the study population, whether multiple SSA country populations were studied, and disease or phenotype. The disease category for each publication was assigned for a range of studies including associated variants, pharmacokinetics of treatment, and diagnosis. Data were entered into SPSS version 20 (Chicago, USA) for analysis.

Summaries for qualitative variables were presented using frequencies and proportions. The association between affiliation of first author with an African institution (AIAFA status) and variables was tested using Chi-square tests. The countries of population studied were organized into four groups—Southern Africa, Eastern Africa, West Africa, and Central Africa—and publications with populations from at least two regions. SSA countries usually were classified into more than one region, meaning Zambia, Zimbabwe, and Mozambique, were classified as Southern Africa while Sudan, Malawi, Rwanda, and Burundi were classified as East Africa. A logistic regression was used to determine odds ratios and confidence intervals. Univariate logistic regression was done to determine unadjusted odds ratios, followed with multivariable regression for adjusted odds ratios. Only variables with *p* values <20% on univariate regression and Chi-square tests were included in the multivariable regression. The level of significance for all tests was 5%.

## Results

There were 508 publications that met the criteria for selection. There was a general increase in the number of articles published. In 2012, a total of 76 published articles was more than double the 2004 total of 32 articles. Characteristics of the publications analyzed are shown in Table 1. The most commonly studied population was South Africa, accounting for 31.1%, followed by Ghana (10.6%), Kenya (7.5%), Gambia (6.5%), Gabon (6.5%), Sudan (5.3%), and Nigeria (5.3%). Other SSA countries (not shown) studied include Tanzania (4.3%), Uganda (4.3%), Burkina Faso (3.3%), Zambia (2.8%), and Cameroon (2.6%).

**Table 1:** Frequency distribution of characteristics of publications

Variable	Frequency	%
Country^a^		
South Africa	158	31.1
Ghana	54	10.6
Kenya	38	7.5
Gambia	33	6.5
Nigeria	27	5.3
Sudan	27	5.3
Region		
Southern Africa	182	35.8
East Africa	123	24.2
West Africa	151	29.7
Central Africa	24	4.3
2 or more regions	30	5.9
Author affiliation		
First author from SSA institution	238	46.9
No author from SSA institution	45	8.9
Others (At least one author from SSA institution but not first author)	225	44.2
Affiliation of first author (*n*=238)		
University	190	79.8
Research institute	40	16.8
Others (Ministry of Health, State Hospital, NGO)	8	3.4
Disease studied^b^		
HIV	92	18.1
Malaria	103	20.3
TB	39	7.7
Cancer	31	6.1
Cardiovascular disease	22	4.3

a Only those countries with at least 5% proportion shown.

b These five diseases were selected because of their high relative frequency in the sample and importance. Several other diseases and phenotypes constituted very small numbers and are not presented. Also, some publications focused on more than one disease.

Regionally, more than one-third (35.8%) of the studies were conducted in a Southern African country, followed by 29.7% in West Africa, 24.2% in East Africa, and 4.3% in Central Africa. The remainder (5.9%) was conducted using data from more than one region of SSA. In 7.9% of the publications, populations from two or more SSA countries were studied. Most studies were about malaria (20.3%), HIV (18.1%), and tuberculosis (7.7%), which accounted for 46.1% of all publications. About one-tenth of the studies were related to chronic NCDs, such as cancer (6.1%), and cardiovascular diseases (CVDs), including hypertension (4.3%).

**Fig. 2:**
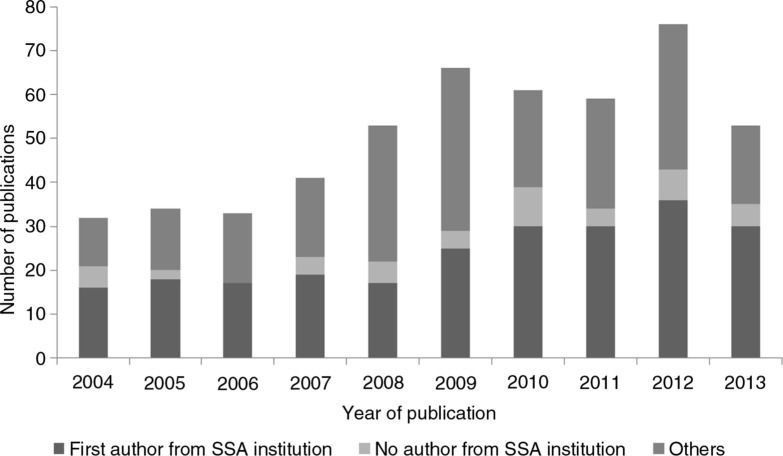
Trends in number of genomic epidemiology publications with author affiliated with an African institution.

The majority of the publications (91.1%) had at least one author affiliated with an African institution while less than half (238, 46.9%) had a first author from an African institution. Among the 238 articles with an African first author, over three-quarters (79.8%) belonged to a university or medical school, 16.8% were affiliated with a research institute, and 3.4% had affiliations with other institutions. Among Southern African publications, 93.6% were from a university or medical school, while less than one-tenth were from research institutes (4.3%) or other organizations (2.1%) (data not shown). However, among East African publications, 68.6% were from universities and 31.4% from research institutes. In West and Central Africa, 55% of authors had affiliations with universities, 36.7% with research institutes, and 8.3% with other organizations. Figure 2 shows the trends in the proportion of publications with an African institution–affiliated first author between 2004 and 2013. There was no evidence of a proportion increase within the 10-year period (*p*=0.331). In fact, there appeared to be a reduction in the proportion of African first authorship between 2007 and 2010 when this proportion rose again.

Table 2 shows the association between African institution first-author affiliation and selected variables. Publications from Southern African countries had significantly higher proportions with AIAFA compared to other regions of Africa (*p*<0.001). An AIAFA was less commonly found among publications involving research participants from more than one SSA country (*p*<0.001). Significantly lower proportions of HIV- and malaria-related articles had an AIAFA, but there was no significant association for TB-related publications. The pattern was reversed for NCDs, such as CVDs and cancer. According to the multiple logistic regression analysis, publications involving Southern African populations were at least four times more likely than those from other SSA regions to have an AIAFA. The odds of an AIAFA were significantly lower among HIV-related publications (OR=0.3, 95% CI=0.16–0.54).

**Table 2:** Cross-tabulations and multivariable logistic regression of African institution–affiliated first author and variables

	Cross-tabulations			Logistic regression analysis	
		
Variable^a^	N	% AIAFA	*P*	Unadjusted OR (95% CI)	Adjusted OR (95% CI)
Region					
Southern Africa (ref)	182	78.0	<0.001	1	1
East Africa	123	28.5		0.11 (0.07–0.19)^b^	0.12 (0.07–0.20)^b^
West Africa	151	31.1		0.13 (0.08–0.21)^b^	0.11 (0.06–0.19)^b^
Central Africa	22	45.5		0.24 (0.10–0.58)^b^	0.24 (0.09–0.62)^b^
2 or more regions	30	13.3		0.04 (0.01–0.13)^b^	0.03 (0.01–0.19)^b^
Number of countries					
1	468	49.1	<0.001	3.87 (1.74–8.57)^b^	1.21 (0.31–4.73)
2 or more (ref)	40	20.0		1	1
HIV related					
Yes	92	37.0	0.036	0.61 (0.38–0.97)^b^	0.30 (0.16–0.54)^b^
No (ref)	416	49.0		1	1
Malaria related					
Yes	103	27.2	<0.001	0.35 (0.22–0.56)^b^	0.61 (0.35–1.07)
No (ref)	405	51.9		1	1
TB related					
Yes	39	51.3	0.564		
No	469	46.5			
Cancer related					
Yes	31	64.5	0.042	2.16 (1.01–4.61)^b^	1.07 (0.44–2.63)
No (ref)	477	45.7		1	1
Cardiovascular diseases					
Yes	22	68.2	0.040	2.53 (1.01–6.31)^b^	1.02 (0.34–3.01)
No (ref)	486	45.9		1	
Year of publication				1.02 (0.96–1.09)	1.04 (0.97–1.12)

a Ref – Reference category for logistic regression.

b Significant at 5% level of significance.

## Discussion

Research output and authorship can serve as a veritable proxy for assessing research capacity of organizations, and to our knowledge, this study is the first to use evidence from genomics publications across SSA to assess the genomic epidemiology research capacity of scientists in the region. Previous studies have focused on biomedical publications in general (22) or those specific to diseases such as HIV (23). A previous study (24) reviewed genomics publications in Cameroon but focused on the ethical issues related to the use of African samples by foreign medical researchers.

This study has shown wide disparities in research output in SSA with a skew toward higher output from Southern Africa, where a majority of the publications originated. South Africa had about three times more publications than any other SSA country during the period. Furthermore, individuals affiliated with a South African university authored an overwhelming majority of publications from South Africa. This is a remarkable finding and an indication of the level of development of genomics research in the country. The high research output from South African authors has been previously reported. For example, Hofman et al. (25), in a study of the health research output of SSA, found that South Africa contributed 40% of all publications. Similarly, a 2007 study showed that South Africa, Egypt, and Nigeria accounted for 60% of Africa's biomedical publications (18).

South Africa's high genomic epidemiology research output reflects the giant strides already taken by this country in developing its biotechnology industry (26–29). Gambia, Kenya, and Ghana have a relatively high number of publications. However, a significant number of these publications are from investigators affiliated with research institutes in those countries. In Gambia, for example, the MRC laboratories funded by the United Kingdom have generated several publications, and a researcher at the organization led the continent-wide publication of a genome-wide association study of malaria (30). The work environment in research institutes seems to provide better support for meaningful research due to funding. For example, Smith et al. (31) showed a much higher use of electronic resources among African research institutes compared with the reliance on textbooks by researchers in teaching hospitals. While SSA-based research institutes funded by organizations in Europe and America have contributed to the research output in SSA countries where they are located, there are potential conflicts of donor versus country research priorities and agenda. In some situations, the most important diseases of public health interest are at risk of being given much less attention, thus denying the local population the potential benefits of genomics research, such as new technologies. These concerns have been expressed by other authors and raised important ethical questions around the choice and focus of local research (24).

Less than one-tenth of the publications used multi-country populations or participants from more than one region. Several authors have advocated for more collaboration between scientists on the African continent, especially for conditions such as HIV, TB, and malaria, which have a high disease burden on the continent (32, 33). An option for encouraging genomics research collaboration is to adopt the Consortium for Advanced Research and Training (CARTA) model, whereby PhD students from several SSA countries are networked by bringing them together periodically while they remain in their home institutions to fulfill their academic requirements (34). Other recent veritable capacity-building initiatives include the Developing Excellence in Leadership, Training and Science Initiative (DELTAs) (35), and H3ABioNet (36).

Although CARTA is focused on population and public health, similar consortia could be devoted to genomics training and include postgraduate training targeting clinicians and other life scientists such as microbiologists, computer scientists, and social scientists.

The finding that almost half of all studies investigated HIV, TB, or malaria is consistent with the huge disease burden of these diseases in SSA. However, less than one-tenth of publications related to cancer or CVDs. There has been a steady rise in the incidence of NCDs, such as cancer, with an even higher projected burden in the next 25 years (3). Hence, greater efforts need to be directed toward research into diagnostic and treatment technologies for conditions such as cancer and CVDs. It is encouraging, however, that H3Africa recently awarded grants for genome epidemiology studies into NCDs (18, 37).

Concerning author affiliation, although a majority had at least one author with an African institution affiliation, less than half were first authors. In fact, in most studies without an African first author, the local authors are mostly involved in the organization and general administrative roles and rarely in the conceptualization, design, data analysis, or writing of the manuscripts. The implications of this finding are twofold. First, the opportunity to build research capacity is lost. Second, SSA communities are denied the chance to have a vibrant genomics research hub that will investigate issues of local relevance. For example, research into the pharmacokinetics of antiretroviral therapy might be more important to a community than work that focuses on the genetic determinants of HIV susceptibility.

This study found no significant increase in the proportion of African first author publications during the 10-year period. In fact, there was a decrease between 2007 and 2009. It is anticipated that this pattern will change with the recent H3Africa's awards, but there is also concern that the steady ‘brain drain’ in developed countries will continue to rob SSA institutions of its youngest and brightest investigators. We expect that this publication will serve as a useful baseline for the evaluation of H3Africa's efforts and successes in the future. Almost 9% of published studies used data from SSA populations but did not include an African-affiliated first author. This observation leads to ethical questions about whether the continent is being denied opportunities to develop capacity. It could be argued that most data are now in the public domain and that any scientist should be able to use data from any other population. Nevertheless, this study, which excluded all publications and relied solely on public domain data, shows that nearly one-tenth of the research involved entirely foreign authors.

African institution first author status was significantly more common in studies involving Southern Africa populations and less common among HIV-related publications. This pattern remained even after adjusting for the year of study and other disease conditions on a multiple logistic regression. The higher odds of AIAFA from Southern African studies appear to be related to the presence of genomics education, training, and research conducted the nation's universities (as the case for South Africa), and not by organizations supported by foreign donors. It is unlikely there can be real developments in a nation's genomics capacity with predominance of foreign-funded institutions conducting genomics research. Less than one-third of publications in West, Central, and East Africa had African first-authored publications, and authors in locally based research institutes produced almost one-third of the publications in these regions. There is an urgent need for capacity building for genomics research in these regions. In most SSA countries, except South Africa, genomics training is hardly done in the universities. Developing genomics training in universities or similar institutions will allow greater involvement by local scientists and foster the capacity of university academics to participate in genomics research (38). In addition, there is a higher likelihood that this effort will encourage the development of research agendas that focus on needs of the native country and prepare the continent for the introduction of personalized medicine that will require well-established genomics education and research.

The current situation in several SSA countries, where up to one-third of AIAFA studies are in foreign-funded local research institutes, supports the case for investing in genomics capacity building in SSA-owned organizations. This investment is especially needed for SSA universities, where there are more opportunities for both undergraduate and postgraduate training and research. Currently, there are several challenges to conducting high-quality research in SSA academic institutions. These include poor power supply, poor internet connectivity, lack of access to full text journal articles (39), publication in low-impact journals (40), and lack of infrastructure for genomics research, including computer laboratories, network computers, and IT support (14).

African countries should take ownership of their own development, and investments in science and technology will yield high dividends in the future. Investments by countries should be matched by genomics research–funding bodies. In addition, there is the need to take the opportunity of initiatives, such as the H3Africa, and provide special funds toward supporting the weak genomics education and research infrastructure in SSA universities. In particular, ensuring access to full text articles and laboratories for hands on wet laboratory experience appear to be an urgent need. Recently, two major publishers—Elsevier and Springer—withdrew access to their journals from the Health Inter-Network Access to Research Initiative (HINARI) network, an initiative that allows researchers in developing countries to access full text journal articles (41). Funds for genomics research could assist in assuaging the effect of this major setback by ensuring partial or total access to journals. In addition, granting bodies should support local researchers to ensure their research is published as open access and available to local scientists. H3Africa has awarded funds for the establishment of collaborative centers (18) located in select SSA universities. Part of the mandate of these centers is to build local research capacity. As much as possible, these centers should actively engage researchers affiliated with those universities and not just conduct high-quality research in an isolated environment. Perhaps a starting point could be the engagement of all scientists in those universities who are currently involved or have been involved in genomics research at some time in the past. In addition, the collaborative centers need to create and sustain networks of academics in related departments such as computer science, bioinformatics, information and communication technology, and the basic biological and life sciences to form genomics and bioinformatics research groups.

Investment into genetics education at all levels has been advocated (3, 42), and this should be given topmost priority in SSA universities. The ongoing effort to reform curricula in SSA through the NIH-funded Medical Education Partnership Initiative (MEPI) funding mechanism, which pairs a US-based academic center with one or two universities in SSA, is an important commitment to enhancing the education in medical schools and during residency training program that will include genomics (43). Collaboration between SSA-based universities involved in the MEPI program provides a unique opportunity to create regional centers of excellence that will promote advances in medicine that includes genomics.

Interdisciplinary collaboration that brings professionals from the biological, clinical, bioinformatics, and computational aspects of genomics training to design best approaches for the teaching of genomics and bioinformatics at undergraduate and postgraduate level are also urgently needed (44). It is also important to involve healthcare providers who may not be genomics researchers but who, in practice, encounter patients who need counseling and/or referral for specialist genetics services (45, 46).

HIV-related publications are another independent variable associated with first authorship in our study. It is unclear why comparatively few AIAFA publish in HIV publications. One explanation is that HIV-related studies are given substantially more foreign support. Alternatively, since it is usually expected that the lead author of any study will be one of the researchers who conceived and initiated the study, the higher number of foreign lead authors reflects the higher proportion of foreign collaborators. Nevertheless, the relatively higher burden of HIV/AIDS in SSA compared to any other region of the world requires a higher degree of lead authorship by authors in this region.

This study has a number of limitations. First, the affiliation of the authors used in this study was established entirely from the information provided in the publication, which might not be completely accurate. In addition, some authors’ affiliations may have changed over the years (due to change of jobs, migration to a different continent, and so on), which could then result in an underestimation or overestimation of present status of first authorship. Second, it is difficult to ascertain the level of collaboration or support offered by the foreign-funded research institutes to the SSA universities in terms of research or teaching. Hence, the conclusions about a probable low level of contribution of institutes to local genomics research and development might not be entirely correct. Third, the exclusion of local journals could have led to an underestimation of first authorship. A recent study showed the relatively low patronage of foreign journals by SSA investigators (40). However, given that the field of genomics is relatively recent and highly specialized, it is unlikely that a significant number of articles were missed. Finally, in assessing the factors influencing first authorship and adjusting for potential confounders, our analysis was limited to the variables that were extracted from the publications.

The strengths of this study include the relatively large number of publications reviewed over a 10-year period. In addition, contrary to similar studies relying on first authors to search articles for review in online databases (25), this study examined all publications in the period of investigation and obtained data on the affiliation of all authors.

## Conclusion

Overall, this study has shown that recent efforts to build genomics capacity in Africa must take in consideration the disparities in geographical and institutional capacities for genomics research, and focus more on locally owned institutions, especially those offering undergraduate and postgraduate training in addition to research. African governments need to provide a more conducive and sustainable research environment in government-owned local institutions that will offer local researchers greater opportunities for genomics research capacity building.

## Supplementary Material

Building local capacity for genomics research in Africa: recommendations from analysis of publications in Sub-Saharan Africa from 2004 to 2013

## Paper context

Genomics has a huge potential to improve diagnosis and treatment of several medical conditions in SSA. However, infrastructure and personnel to conduct genomics research in the region are suboptimal.

We examined the author affiliations of genomic epidemiology publications in SSA and found significant disparities between SSA countries in genomics research capacity. There is an urgent need to focus on developing local capacity, especially those affiliated with SSA universities.
