# Supplementary material for: Building local capacity for genomics research in Africa: recommendations from analysis of publications in Sub-Saharan Africa from 2004 to 2013
Source: Glob Health Action. 2016 May 12;9:10.3402/gha.v9.31026. doi: 10.3402/gha.v9.31026 (PMC4867048; doi:10.3402/gha.v9.31026)
Supplement: Building local capacity for genomics research in Africa: recommendations from analysis of publications in Sub-Saharan Africa from 2004 to 2013 [file GHA-9-31026-s001.doc]

**Received:** 2016-01-17, Revised: 2016-03-10, Accepted: 2016-03-10, Published: xx

**Title: Building Local Capacity for Genomics Research in Africa: Recommendations from analysis of publications in Sub-Saharan Africa from 2004 to 2013**

**Running title:** Building African genomics research capacity

**Authors:**

Babatunde O. Adedokun^1*^, Christopher O. Olopade^2^, Olufunmilayo I. Olopade^3^

^1^ Department of Epidemiology and Medical Statistics, College of Medicine, University of Ibadan, Ibadan, Nigeria

email: [tukankar@yahoo.co.uk](mailto:tukankar@yahoo.co.uk)

^2^ Centre for Global Health, Department of Medicine, University of Chicago, Chicago IL 60637, USA

email: [solopade@bsd.uchicago.edu](mailto:solopade@bsd.uchicago.edu)

^3^ Center for Clinical Cancer Genetics and Global Health, Department of Medicine, University of Chicago, Chicago IL 60637, USA

email: [folopade@medicine.bsd.uchicago.edu](mailto:folopade@medicine.bsd.uchicago.edu)

***Corresponding author**

Department of Epidemiology and Medical Statistics, College of Medicine, University of Ibadan, Ibadan, Nigeria

Email: [tukankar@yahoo.co.uk](mailto:tukankar@yahoo.co.uk)

**Word count**: Abstract: 256; Manuscript: 3296

**Keywords**: Capacity building, genomics, health research, sub-Saharan Africa, bibliometric analysis

**Abstract:**

**Background**: The poor genomics research capacity of Sub-Saharan Africa (SSA) could prevent maximal benefits from the applications of genomics in the practice of medicine and research. The objective of this study is to examine the author affiliations of genomic epidemiology publications in order to make recommendations for building local genomics research capacity in SSA.

**Methods**: Sub-Saharan African genomic epidemiology articles published in the period between 2004 and 2013 were extracted from the Human Genome Epidemiology (HuGE) database. Data on authorship details, country of population studied, and phenotype or disease were extracted. Factors associated with first author with a SSA institution affiliation (AIAFA) were determined using Chi square test and multiple logistic regression analysis.

**Results**: The most commonly studied population was South Africa, accounting for 31.1%, followed by Ghana (10.6%) and Kenya (7.5%). About one tenth of the papers were related to non-communicable diseases such as cancer (6.1%) and cardiovascular diseases (4.3%). Less than half of first authors (46.9%) were affiliated with an African institution. Among the 238 articles with an African first author, over three quarters (79.8%) belonged to a university or medical school, 16.8% were affiliated with a research institute and 3.4% had affiliations with other institutions.

**Conclusions**: Significant disparities currently exist between SSA countries in genomics research capacity. South Africa has the highest genomics research output, which is reflected in the investments made in its genomics and biotechnology sector. These findings underscore the need to focus on developing local capacity, especially among those affiliated with SSA universities where there are more opportunities for teaching and research.

**Background**

Genomic medicine has experienced astronomical growth in recent years (1,2). The field of genomics holds great promise for health care and medical research, as identification of the genetic determinants of disease or other phenotypes will bring about significant improvements in diagnosis, prevention and treatment of several disease conditions (3,4). Genomics is particularly attractive for Sub-Saharan African countries where new technologies and products from genomics research can help mitigate the heavy burden of infectious and chronic non-communicable diseases (5). Additionally, such genomics research should be culturally acceptable to Sub-Saharan African populations (6,7).

In spite of these exciting developments, there are fears that already existing inequalities in health care access will only worsen as technologies and discoveries resulting from genomics research remain affordable only to those who reside in more developed countries (3,8). Furthermore, genomics research is likely to be biased towards therapeutic and diagnostic applications for conditions affecting populations in wealthy countries with little or no benefit for most people in low- to middle-income countries (3). There is thus an urgent need to invest in capacity building and infrastructure development and to encourage investments by SSA governments into genomics research (5, 9 – 13). Such efforts will enable less dependence on the market-driven research agenda of the developed world for the health needs of low- to middle-income countries (3).

Currently, the state of infrastructure and capacity of SSA scientists is poor (14,15). Additionally, the current investments by governments in SSA remain very poor. In most countries in SSA, health research is allocated less than 0.5% of the national health budget (16). Genomics research is a promising area , and deserves greater attention by African governments (5). In response to the challenge of poor research funding in Africa, the Human Heredity and Health (H3Africa) initiative, jointly funded by the National Institutes of Health (NIH) and the Wellcome Trust, recently awarded several millions of dollars in grants to investigators on the African continent (17, 18, 19).

While the H3Africa efforts hold great promise for the transformation of genomics research in Africa through capacity building and better research facilities, there is a need to document the state of local or regional genomics research productivity in order to guide equitable distribution of resources. Presently, there are few studies that have examined the current local capacity of SSA scientists for genomics research. Without data on SSA local and regional capacity, activities of the H3Africa projects and other similar interventions to build local and regional research capacity in SSA could be jeopardized. The objective of this review is to examine existing capacity through author affiliations of genome epidemiology publications in order to make recommendations for local and regional genomics research capacity building in SSA.

**Methods**

We extracted and analyzed Genomic epidemiology publications with SSA study populations over a ten-year period using the HuGE Pub Lit database. Sub-Saharan Africa, as defined for the purpose of this review, includes Sudan, which is excluded from the United Nations (UN) definition of SSA but included by UN agencies. The articles used were obtained from the Human Genome Epidemiology (HuGE) published literature database (HuGE Pub Lit). The HuGe Pub Lit was launched in 2001 to track publications related to human genome epidemiology (20). Articles in the database include studies of human populations that have been published since October 2000 and have English-language abstracts. In addition, the HuGE Pub Lit includes only publications where genotypes must have been measured at one or more loci while gene discovery articles, such as linkage analysis and gene mapping for high-risk families, were excluded.

For this analysis, 508 articles published between January 2004 and December 2013 were selected after excluding publications that used data from a foreign population in addition to a SSA population in the same study, and those using data on SSA born individuals residing in Europe and America (Figure 1, see supplemental for full details). The selection of articles is presented according to PRISMA (Preferred Items for Systematic Reviews and Meta-analysis) guidelines (21). The publications were excluded because research conducted exclusively on African participants would allow better assessment of local research capacity. One article written in the Russian language was excluded. Studies using data from only SSA populations were chosen in order to adequately assess the contribution of local SSA scientists to genomics research involving local participants. Information extracted from each of the 508 articles include: year of publication, affiliation of first author, presence of any African author and affiliation of first author if from a SSA institution. In cases where there were multiple affiliations for an author, such an author was still classified as being affiliated with SSA as long as there was a SSA institution among the affiliations. Other variables extracted include country of population studied, whether multiple SSA country populations were studied, phenotype or disease. The disease category for each publication was assigned for a range of studies including associated variants, pharmacokinetics of treatment and diagnosis. Data was entered into SPSS version 20 (Chicago, USA) for analysis.

Summaries for qualitative variables were presented using frequencies and proportions. Association between publications’ African Institution affiliated first author (AIAFA) status and variables was tested using Chi square tests. Country of population studied was organized into four groups – Southern Africa, Eastern Africa, West Africa, Central Africa – and publications with populations from at least two regions. Concerning those SSA countries usually classified to more than one region, Zambia, Zimbabwe and Mozambique were classified as Southern Africa while Sudan, Malawi, Rwanda and Burundi were classified as East Africa. Logistic regression was used to determine odds ratios and confidence intervals. Univariate logistic regression was done to determine unadjusted odds ratios, followed with multivariable regression for adjusted odds ratios. Only variables with p values <20% on univariate regression and Chi-square tests were included in the multivariable regression. Level of significance for all tests was 5%.

[ INSERT FIGURE 1]

**Results**

There were 508 publications that met the criteria for selection. There was a general increase in the number of articles published, with the number in 2012 (76 articles) more than doubling that in 2004 (32 articles). Characteristics of the publications analyzed are shown in Table 1. The most commonly studied population was South Africa, accounting for 31.1%, followed by Ghana (10.6%), Kenya (7.5%), Gambia (6.5%), Gabon (6.5%), Sudan (5.3%), and Nigeria (5.3%). Other SSA countries (not shown) studied include: Tanzania (4.3%), Uganda (4.3%), Burkina Faso (3.3%), Zambia (2.8%), and Cameroon (2.6%).

Regionally, more than a third (35.8%) of the studies were conducted in a Southern African country, followed by 29.7% in West Africa, 24.2% in East Africa, and 4.3% in Central Africa. The remainder (5.9%) was conducted using data from more than one region of SSA. In 7.9% of publications, populations from two or more SSA countries were studied. Most studies were about malaria (20.3%), HIV (18.1%), and tuberculosis (7.7%), which accounted for 46.1% of all publications. About one tenth of the studies were related to chronic non-communicable diseases such as cancer (6.1%) and cardiovascular diseases, including hypertension (4.3%).

[INSERT TABLE 1]

Majority of the publications (91.1%) had at least one author affiliated with an African institution while less than half (238, 46.9%) had its first author from an African institution. Among the 238 articles with an African first author, over three quarters (79.8%) belonged to a university or medical school, 16.8% were affiliated with a research institute and 3.4% had affiliations with other institutions. Among Southern African publications, 93.6% were from a university or medical school while less than a tenth were from research institutes (4.3%) or other organizations (2.1%) (data not shown). However, among East African publications, 68.6% were from universities and 31.4% from research institutes. In West and Central Africa, 55% of authors had affiliations with universities, 36.7% with research institutes and 8.3% with other organizations. Figure 2 shows the trends in the proportion of publications with an African institution-affiliated first author between 2004 and 2013. There was no evidence of a proportion increase within the ten-year period (p=0.331). In fact, there appeared to be a reduction in the proportion of African first authorship between 2007 and 2010 when this proportion rose again.

[INSERT FIGURE 2]

Table 2 shows the association between African institution first author affiliation and selected variables. Publications from Southern African countries had significantly higher proportions with AIAFA compared to other regions of Africa (p<0.001). An AIAFA was less commonly found among publications involving research participants from more than one SSA country (p<0.001). Significantly lower proportions of HIV- and malaria-related articles had an AIAFA, but there was no significant association for TB-related publications. The pattern was reversed for NCDs such as CVD and cancer. According to the multiple logistic regression analysis, publications involving Southern African populations were at least four times more likely than those from other SSA regions to have AIAFA. The odds of AIAFA were significantly lower among HIV-related publications (OR = 0.3, 95% CI = 0.16 – 0.54).

[INSERT TABLE 2]

**Discussion**

Research output and authorship can serve as a veritable proxy for assessing research capacity of organizations, and to our knowledge, this study is the first to use evidence from genomics publications across SSA to assess genomic epidemiology research capacity of scientists in the region. Previous studies have focused on biomedical publications in general (22) or those specific for diseases such as HIV (23). A previous study (24) reviewed genomics publications in Cameroon, but focused on ethical issues related to use of African samples by foreign medical researchers.

This study has shown wide disparities in research output in SSA with a skew towards higher output from Southern Africa, where a majority of the publications originated. South Africa had about three times more publications than any other SSA country during the period. Furthermore, individuals affiliated with a South African university authored an overwhelming majority of publications from South Africa. This is a remarkable finding and an indication of the level of development of genomics research in the country. The high research output from South African authors has been previously reported. For example, Hofman et al (25) in a study of health research output of SSA found that South Africa contributed 40% of all publications. Similarly, a 2007 study showed that South Africa, Egypt and Nigeria accounted for 60% of Africa’s biomedical publications (18).

South Africa’s high genomic epidemiology research output reflects the giant strides already taken by this country in developing its biotechnology industry (26 – 29). Gambia, Kenya and Ghana have a relatively high number of publications. However, a significant number of these publications are from investigators affiliated with research institutes in those countries. In Gambia, for example, the MRC laboratories funded by the United Kingdom have generated several publications, and a researcher at the organization led the continent-wide publication of a genome-wide association study of malaria (30). The work environment in research institutes seems to provide better support for meaningful research due to funding. For example, Smith et al (31) showed much higher use of electronic resources among African research institutes compared to the reliance on textbooks for researchers in teaching hospitals. While SSA-based research institutes funded by organizations in Europe and America have contributed to research output in SSA countries where they are located, there are potential conflicts of donor versus country research priorities and agenda. In some situations, the most important diseases of public health interest could be given much less attention, thus denying the local population the potential benefits of genomics research such as new technologies. These concerns have been expressed by other authors and raised important ethical questions around the choice and focus of local research (24).

Less than a tenth of publications used multi-country populations or participants from more than one region. Several authors have advocated for more collaboration between scientists on the African continent, especially for conditions such as HIV, TB and malaria, which have a high disease burden on the continent (32, 33). An option for encouraging genomics research collaboration is by adopting the Consortium for Advanced Research and Training (CARTA) model whereby PhD students from several SSA countries are networked by bringing them together periodically while they remain in their home institutions to fulfill their academic requirements (34). Other recent veritable capacity building initiatives include the Developing Excellence in Leadership, Training and Science Initiative (DELTAs) (35) and H3ABioNet (36).

While CARTA is focused on population and public health, similar consortia could be devoted to genomics training and include postgraduate training targeting clinicians, other life scientists such as microbiologists, computer scientists and social scientists.

The finding that almost half of all studies investigated HIV, TB or malaria is consistent with the huge disease burden of these diseases in Sub-Saharan Africa. However, less than a tenth of publications were related to cancer or CVDs. There has been a steady rise in the incidence of non-communicable diseases such as cancer with an even higher projected burden in the next 25 years (3). Hence, greater efforts need to be directed towards research into diagnostic and treatment technologies for conditions such as cancer and CVDs. It is encouraging, however, that H3Africa recently awarded grants for genome epidemiology studies into NCDs (18, 37).

Concerning author affiliation, though a majority had at least one author with an African institution affiliation, less than half were first authors. In fact, in most studies without an African first author, the local authors are mostly involved in the organization and general administrative roles and rarely in the conceptualization, design, data analysis or writing of the manuscript. The implications of this finding are two-fold. First, the opportunity to build research capacity is missed, and secondly, SSA communities are denied the chance to have a vibrant genomics research hub that will investigate issues of local relevance. For example, research into pharmacokinetics of antiretroviral therapy might be more important to a community than that focusing on the genetic determinants of HIV susceptibility.

This study found no significant increase in the proportion of African first author publications during the ten-year period. In fact, there was a decrease between 2007 and 2009. It is anticipated that this pattern will change with the recent H3Africa’s awards, but there is also concern that the steady ‘brain drain’ in developed countries will continue to rob SSA institutions of its youngest and brightest investigators. We expect that this publication will serve as a useful baseline for evaluation of H3Africa’s efforts and successes in the future. The observation that almost 9% of studies that were published using data from SSA populations did not include an African-affiliated first author raises ethical questions, as this trend denies the continent the opportunities for capacity development. It could be argued that most data are now in the public domain and that any scientist should be able to use data from any other population. However, this study excluded all publications and solely used public domain data, and yet, nearly a tenth involved entirely foreign authors.

African institution first author status was significantly more common in studies involving Southern Africa populations and less common among HIV-related publications. This pattern remained even after adjusting for year of study and other disease conditions on multiple logistic regression. The higher odds of AIAFA from Southern African studies appear to be related to the presence of genomics education, training and research by the nation’s universities (as the case for South Africa), and not by organizations supported by foreign donors. It is unlikely there can be real developments in a nation’s genomics capacity with a predominance of foreign funded institutions conducting genomics research. Less than a third of publications in West, Central and East Africa had African first-authored publications, and authors in locally based research institutes produced almost a third of the publications in these regions. There is an urgent need for capacity building for genomics research in these regions. In most SSA countries except South Africa, genomics training is hardly done in the universities. Developing genomics training in universities or similar institutions will allow greater involvement of local scientists and foster capacity of university academics to participate in genomics research (38). Additionally, there is a higher likelihood that this effort will encourage the development of research agendas that will focus on needs of the native country and prepare the continent for the era of personalized medicine that will require well established genomics education and research.

The current situation in several SSA countries where up to a third of AIAFA studies are in foreign funded local research institutes support the case for investing in genomics capacity building in SSA-owned organizations. This investment is especially needed for SSA universities where there are more opportunities for both undergraduate and postgraduate training and research. Currently, there are several challenges with conducting high quality research in SSA academic institutions. These include poor power supply, poor internet connectivity, lack of access to full text journal articles [39] and publication in low impact journals (40), and lack of infrastructure for genomics research including computer laboratories, network computers and IT support (14).

African countries should take ownership of their own development, and investments in science and technology will yield high dividends in the future. Investments by countries should be matched by genomics research funding bodies. In addition, there is the need to take the opportunity of initiatives, such as the H3Africa, to provide special funds towards supporting the weak genomics education and research infrastructure in SSA universities. In particular, ensuring access to full text articles and laboratories for hands on wet laboratory experience appear to be an urgent need. Recently, two major publishers – Elsevier and Springer - withdrew access to their journals from the Health Inter-Network Access to Research Initiative (HINARI) network, an initiative that allows researchers in developing countries to access full text journal articles (41). Funds for genomics research could assist in assuaging the effect of this major setback by ensuring partial or total access to journals. Additionally, granting bodies should support local researchers to ensure their research is published as open access and available to local scientists. H3Africa has awarded funds for the establishment of collaborative centers (18) located in select SSA universities. Part of the mandate of these centers is to build local research capacity. As much as possible, these centers should actively engage researchers affiliated with those universities and not just conduct high quality research in an isolated environment. Perhaps a starting point could be the engagement of all scientists in those universities who are currently involved or have been involved in genomics research at some time in the past. In addition, the collaborative centers need to create and sustain networks of academics in related departments such as Computer Science, Bioinformatics, Information and Communication Technology, and the basic biological and life sciences to form genomics and bioinformatics research groups.

Investment into genetics education at all levels has been advocated (3, 42) and this should be given topmost priority in SSA universities. The ongoing effort to reform curricula in SSA through the NIH-funded Medical Education Partnership Initiative (MEPI) funding mechanism, which pairs a US-based academic center with one or two universities in SSA, is an important commitment to enhancing the education in medical schools and during residency training program that will include genomics (43). Collaboration between SSA-based universities involved in the MEPI program provides a unique opportunity to create regional centers of excellence that will promote advances in medicine that includes genomics.

Interdisciplinary collaboration that brings professionals from the biological, clinical, bioinformatics, and computational aspects of genomics training to design best approaches for teaching of genomics and bioinformatics at undergraduate and postgraduate level are also urgently needed (44). Engagement of healthcare providers who may not be involved in genomics research but in their routine practice will come across patients who will need counseling and/or referral for specialist genetics services is also important (45, 46).

Another variable independently associated with first authorship in our study was HIV-related publications. The reasons for the lower odds of AIAFA among HIV publications are unclear. However, it could be due to a higher foreign support for HIV-related studies or because a higher proportion of studies conceived or initiated by foreign collaborators usually come with the expectation that the person who conceived the idea will be the lead author. The relatively higher burden of HIV/AIDS in SSA compared to any other region of the world requires a higher degree of lead authorship by authors in this region.

This study has a number of limitations. First, the affiliation of authors used in this study relied entirely on the information provided in the publication and might not be completely accurate. In addition, some authors’ affiliations could have changed over the years (due to change of jobs, migration to a different continent, etc), which could then result in an underestimation or overestimation of present status of first authorship. Secondly, it is difficult to ascertain the level of collaboration or support offered by the foreign-funded research institutes to the SSA universities in terms of research or teaching. Hence, the conclusions about a probable low level of contribution of institutes to local genomics research and development might not be entirely correct. Thirdly, the exclusion of local journals could have underestimated first authorship. A recent study showed the relatively low patronage of foreign journals by SSA investigators (40). However, given that the field of genomics is relatively recent and highly specialized, it is unlikely that a significant number of articles have been missed. Finally, in assessing the factors influencing first authorship and adjusting for potential confounders, our analysis was limited to the variables that were extracted from the publications.

Strengths of this study include the relatively large number of publications reviewed over a ten-year period. In addition, contrary to similar studies relying on first authors to search articles for review in online databases (25), this study examined all publications in the period of investigation and obtained data on the affiliation of all authors.

**Conclusion**

Overall, this study has shown that recent attempts at building genomics capacity in Africa need to consider the disparities in geographical and institutional capacities for genomics research and focus more on locally owned institutions, especially those offering undergraduate and postgraduate training in addition to research. African governments need to provide a more conducive and sustainable research environment in government owned local institutions that will offer local researchers greater opportunities for genomics research capacity building.

**Paper context:**

Genomics has a huge potential to improve diagnosis and treatment of several medical conditions in sub-Saharan Africa (SSA). However, infrastructure and personnel to conduct genomics research in the region are suboptimal.

We examined the author affiliations of genomic epidemiology publications in SSA and found significant disparities between SSA countries in genomics research capacity. There is an urgent need to focus on developing local capacity, especially those affiliated with SSA universities.

**Authors’ contributions**

BOA and OIO were responsible for the conceptualization of the study. BOA did the extraction of the data while OIO and COO validated a portion of the studies reviewed. All authors participated in the analysis and interpretation of the data and were involved in manuscript writing and final approval of the manuscript.

**Competing interests:**

The authors declare that they have no competing interests.

Acknowledgement

This paper received support from the National Institutes of Health International Partnership for Interdisciplinary Research Training in Chronic Non-Communicable Diseases and Disorders across the Lifespan (D43) training grant number NIH D43 TW009112, and the National Cancer Institute (NCI) grant number NCI CA161032..

**References**

1. Feero WG, Guttmacher AE, Collins FS: Genomic medicine - an updated primer. N Engl J Med 2001–2011, 2010;362.

2. McCarthy JJ, McLeod HL, Ginsburg GS Genomic Medicine: A Decade of Successes, Challenges, and Opportunities [Sci Transl Med.](http://www.ncbi.nlm.nih.gov/pubmed/?term=McCarthy+Genomic+medicine%3A+A+decade+of+successes+challenges) 2013; 5:189sr4.

3. World Health Organization, 2008–2013 Action Plan for the Global Strategy for the Prevention and Control of Non communicable Diseases (WHO, Geneva, 2008); [www.who.int/nmh/Actionplan-PC-NCD-2008.pdf](http://www.who.int/nmh/Actionplan-PC-NCD-2008.pdf).

4. Kumar D. Genomic medicine: a new frontier of medicine in the twenty first century. [Genomic Med.](http://www.ncbi.nlm.nih.gov/pubmed/?term=kumar+genomic+medicine+a+new+frontier+of+medicine+twenty+first) 2007; 1: 3-7.

5. Peprah E, Wonkam A Biomedical research, a tool to address the health issues that affect African populations. Globalization and Health 2013; 9: 50

6. [Jegede AS](http://www.ncbi.nlm.nih.gov/pubmed/?term=Jegede AS%5BAuthor%5D&cauthor=true&cauthor_uid=19671074). Culture and genetic screening in Africa. Dev World Bioeth. 2009 Dec;9(3):128-37. doi: 10.1111/j.1471-8847.2009.00259.x. Epub 2009 Aug 4.

7. Fagbemiro L, Adebamowo C. [Knowledge and attitudes to personal genomics testing for complex diseases among Nigerians.](http://www.ncbi.nlm.nih.gov/pubmed/24766930) BMC Med Ethics. 2014 Apr 27;15:34. doi: 10.1186/1472-6939-15-34.

8. Ramsay M. Africa: Continent of genome contrasts with implications for biomedical research and health. FEBS Letters 2012; 586: 2813–2819

# 9. [Hardy BJ](http://www.ncbi.nlm.nih.gov/pubmed?term=Hardy BJ%5BAuthor%5D&cauthor=true&cauthor_uid=18802418), [Séguin B](http://www.ncbi.nlm.nih.gov/pubmed?term=Séguin B%5BAuthor%5D&cauthor=true&cauthor_uid=18802418), [Goodsaid F](http://www.ncbi.nlm.nih.gov/pubmed?term=Goodsaid F%5BAuthor%5D&cauthor=true&cauthor_uid=18802418), [Jimenez-Sanchez G](http://www.ncbi.nlm.nih.gov/pubmed?term=Jimenez-Sanchez G%5BAuthor%5D&cauthor=true&cauthor_uid=18802418), [Singer PA](http://www.ncbi.nlm.nih.gov/pubmed?term=Singer PA%5BAuthor%5D&cauthor=true&cauthor_uid=18802418), [Daar AS](http://www.ncbi.nlm.nih.gov/pubmed?term=Daar AS%5BAuthor%5D&cauthor=true&cauthor_uid=18802418). The next for genomic medicine: challenges and opportunities for the developing world. [Nat Rev Genet.](http://www.ncbi.nlm.nih.gov/pubmed/?term=Hardy+2008+The+next+steps+for+genomic+medicine%3A+challenges+and+opportunities+for+the+developing+world) 2008 Oct;9 Suppl 1:S23-7

10. Wonkam A, Mayosi BM Genomic medicine in Africa: promise, problems and prospects. Genome Medicine 2014; 6:11

# 11. [**Singer** PA](http://www.ncbi.nlm.nih.gov/pubmed?term=Singer PA%5BAuthor%5D&cauthor=true&cauthor_uid=17703557), [Court EB](http://www.ncbi.nlm.nih.gov/pubmed?term=Court EB%5BAuthor%5D&cauthor=true&cauthor_uid=17703557), [Bhatt A](http://www.ncbi.nlm.nih.gov/pubmed?term=Bhatt A%5BAuthor%5D&cauthor=true&cauthor_uid=17703557), [Frew SE](http://www.ncbi.nlm.nih.gov/pubmed?term=Frew SE%5BAuthor%5D&cauthor=true&cauthor_uid=17703557), [Greenwood H](http://www.ncbi.nlm.nih.gov/pubmed?term=Greenwood H%5BAuthor%5D&cauthor=true&cauthor_uid=17703557), [Persad DL](http://www.ncbi.nlm.nih.gov/pubmed?term=Persad DL%5BAuthor%5D&cauthor=true&cauthor_uid=17703557) et al. **Applying** **genomics-related** **technologies** for Africa's health needs. [Afr J Med Med Sci.](http://www.ncbi.nlm.nih.gov/pubmed/?term=Singer+applying+genomics-related+technologies) 2007; 36 Suppl:7-14.

# 12. [Smith AC](http://www.ncbi.nlm.nih.gov/pubmed?term=Smith AC%5BAuthor%5D&cauthor=true&cauthor_uid=15667651), [Mugabe J](http://www.ncbi.nlm.nih.gov/pubmed?term=Mugabe J%5BAuthor%5D&cauthor=true&cauthor_uid=15667651), [Singer PA](http://www.ncbi.nlm.nih.gov/pubmed?term=Singer PA%5BAuthor%5D&cauthor=true&cauthor_uid=15667651), [Daar AS](http://www.ncbi.nlm.nih.gov/pubmed?term=Daar AS%5BAuthor%5D&cauthor=true&cauthor_uid=15667651). ‘Harnessing genomics to improve health in Africa’ - an executive course to support **genomics** policy. [Health Res Policy Syst.](http://www.ncbi.nlm.nih.gov/pubmed/?term=Smith+2005+harnessing+genomics+to+improve+health+in+Africa) 2005 Jan 24; 3:2.

13. Williams SM, Tishkoff SA. [Exploring genomic studies in Africa.](http://www.ncbi.nlm.nih.gov/pubmed/21745422) Genome Med. 2011 Jul 8; 3:45. doi: 10.1186/gm261

14. Kebede D, Zielinski C, Mbondji PE, Piexoto M, Sanou I, Kouvividila W, Lusamba-Dikassa P Research output of health research institutions and its use in 42 sub-Saharan African countries: results of a review by structured questionnaire. J R Soc Med 2014; DOI: 10.1177/0141076813517681J

15. Nchinda TC Research capacity strengthening in the South Social Science & Medicine. 2002; 54: 1699–1711

16. Lansang MA, Dennis R. Building capacity in health research in the developing world. Bull WHO 2004; 82: 764-770.

17. [H3Africa Consortium](http://www.ncbi.nlm.nih.gov/pubmed/?term=H3Africa Consortium%5BCorporate Author%5D). Research capacity. Enabling the genomic revolution in Africa. Science. 2014 Jun 20;344(6190):1346-8. doi: 10.1126/science.1251546.

18. National Institutes of Health: NIH awards $17 million in grants to augment genomics research in Africa. Downloaded from <http://www.nih.gov/news/health/oct2013/nhgri-18.htm>

19. Adoga MP, Fatumo SA, Agwale SM H3Africa: a tipping point for a revolution in bioinformatics, genomics and health research in Africa. Source Code for Biology and Medicine 2014; 9:10

20. Lin BK, Clyne M, Walsh M, Gomez O, Yu W, Gwinn M, Khoury MJ. [Tracking the epidemiology of human genes in the literature: the HuGE Published Literature database.](http://www.ncbi.nlm.nih.gov/pubmed/16641305) Am J Epidemiol. 2006 Jul 1;164(1):1-4. Epub 2006 Apr 26. Review.

21. Moher D, Liberati A, Tetzlaff J, Altman DG, the PRISMA Group. Preferred reporting items for systematic reviews and meta-analyses: the PRISMA statement. PloS Med 2009. 6(7):e1000097; doi:10.1371/journal.pmed.1000097

22. Uthman OA, Uthman MB Geography of Africa biomedical publications: An analysis of 1996 – 2005 Pubmed papers. Int J Health Geographics 2007; 6:46 doi: 10.1186/1476-072X-6-46

23. Uthman OA Pattern and determinants of HIV research productivity in sub-Saharan Africa: bibliometric analysis of 1981 to 2009 PubMed papers. BMC Infectious Diseases 2010; 10: 47

24. Wonkam A, Kenfack MA, Muna WFT, Ouwe-missi-oukem-boyer O Ethics Of Human Genetic Studies In Sub-Saharan Africa: The Case Of Cameroon through A Bibliometric Analysis. Developing World Bioethics 2011; 11: 120–127dewb

# 25. Hofman KJ, Kanyengo CW, Rapp BA, Kotzin S Mapping the health research landscape in Sub-Saharan Africa: a study of trends in biomedical publications. J Med Libr Assoc. 2009; 97: 41- 44

26. [Hardy BJ](http://www.ncbi.nlm.nih.gov/pubmed?term=Hardy BJ%5BAuthor%5D&cauthor=true&cauthor_uid=18802417), [Séguin B](http://www.ncbi.nlm.nih.gov/pubmed?term=Séguin B%5BAuthor%5D&cauthor=true&cauthor_uid=18802417), [Ramesar R](http://www.ncbi.nlm.nih.gov/pubmed?term=Ramesar R%5BAuthor%5D&cauthor=true&cauthor_uid=18802417), [Singer PA](http://www.ncbi.nlm.nih.gov/pubmed?term=Singer PA%5BAuthor%5D&cauthor=true&cauthor_uid=18802417), [Daar AS](http://www.ncbi.nlm.nih.gov/pubmed?term=Daar AS%5BAuthor%5D&cauthor=true&cauthor_uid=18802417). South Africa: from species cradle to genomic applications. [Nat Rev Genet.](http://www.ncbi.nlm.nih.gov/pubmed/?term=Hardy+2008+south+africa+from+species+cradle+to+genomic+applicatrions) 2008 Oct; 9:S19-23.

27.Warnich L, Drogemoller BI, Pepper MS, Dandara C, Wright EB Pharmacogenomic Research in South Africa: Lessons Learned and Future Opportunities in the Rainbow Nation. Current Pharmacogenomics and Personalized Medicine 2011; 9: 191-207

28. Ndimba BK, Thomas LA. [Proteomics in South Africa: current status, challenges and prospects.](http://www.ncbi.nlm.nih.gov/pubmed/19016510) Biotechnol J. 2008 Nov; 3:1368-74. doi: 10.1002/biot.200800236. Review.

29.Motari M, Quach U, Thorsteinsdottie H, Martin DK, Daar AS, Singer PA South Africa—blazing a trail for African biotechnology Nature Biotechnology 2004; 22: DC37 - 42

# 30. Jallow M, Teo YY, Small KS, Rockett KA, Deloukas P, Clark TG et al Genome-wide and fine-resolution association analysis of malaria in West Africa. Nat Genet 2009; 41:657-665

31. Smith H, Bukirwa H, Mukasa O, Snell P, Adeh-Nsoh S, Mbuyita S et al Access to electronic health knowledge in five countries in Africa: a descriptive study. BMC Health Services Research 2007; 7:72 doi:10.1186/1472-6963-7-72

32. [Collins FS](http://www.ncbi.nlm.nih.gov/pubmed/?term=Collins FS%5BAuthor%5D&cauthor=true&cauthor_uid=21127233), [Glass RI](http://www.ncbi.nlm.nih.gov/pubmed/?term=Glass RI%5BAuthor%5D&cauthor=true&cauthor_uid=21127233), [Whitescarver J](http://www.ncbi.nlm.nih.gov/pubmed/?term=Whitescarver J%5BAuthor%5D&cauthor=true&cauthor_uid=21127233), [Wakefield M](http://www.ncbi.nlm.nih.gov/pubmed/?term=Wakefield M%5BAuthor%5D&cauthor=true&cauthor_uid=21127233), [Goosby EP](http://www.ncbi.nlm.nih.gov/pubmed/?term=Goosby EP%5BAuthor%5D&cauthor=true&cauthor_uid=21127233). Public health. Developing health workforce capacity in Africa. Science. 2010 Dec 3;330(6009):1324-5. doi: 10.1126/science.1199930

33. Mgone CS. [Strengthening of the clinical research capacity for malaria: a shared responsibility.](http://www.ncbi.nlm.nih.gov/pubmed/21144085) Malar J. 2010 Dec 13;9 Suppl 3:S5. doi: 10.1186/1475-2875-9-S3-S5. Review.

34. Ezeh AC, Izugbara CO, Kabiru CW, Fonn S, Kahn K, Manderson L et al Building capacity for public and population health research in Africa: the consortium for advanced research training in Africa (CARTA) model. Global Health Action 2010; 3: 5693 - DOI: 10.3402/gha.v3i0.5693

35. Wellcome Trust. African leaders, international partners launch new initiatives to spur scientific research in Africa. 2015 Available at <http://www.wellcome.ac.uk/News/2015/WTP059734.htm>. Accessed February 27, 2016

36. [Mulder NJ](http://www.ncbi.nlm.nih.gov/pubmed/?term=Mulder NJ%5BAuthor%5D&cauthor=true&cauthor_uid=26627985), [Adebiyi E](http://www.ncbi.nlm.nih.gov/pubmed/?term=Adebiyi E%5BAuthor%5D&cauthor=true&cauthor_uid=26627985), [Alami R](http://www.ncbi.nlm.nih.gov/pubmed/?term=Alami R%5BAuthor%5D&cauthor=true&cauthor_uid=26627985), [Benkahla A](http://www.ncbi.nlm.nih.gov/pubmed/?term=Benkahla A%5BAuthor%5D&cauthor=true&cauthor_uid=26627985), [Brandful J](http://www.ncbi.nlm.nih.gov/pubmed/?term=Brandful J%5BAuthor%5D&cauthor=true&cauthor_uid=26627985), [Doumbia S](http://www.ncbi.nlm.nih.gov/pubmed/?term=Doumbia S%5BAuthor%5D&cauthor=true&cauthor_uid=26627985). H3ABioNet, a sustainable pan-African bioinformatics network for human heredity and health in Africa. Genome Res. 2016 Feb;26(2):271-7. doi: 10.1101/gr.196295.115. Epub 2015 Dec 1.

37. [Owolabi MO](http://www.ncbi.nlm.nih.gov/pubmed/?term=Owolabi MO%5BAuthor%5D&cauthor=true&cauthor_uid=24878536), [Mensah GA](http://www.ncbi.nlm.nih.gov/pubmed/?term=Mensah GA%5BAuthor%5D&cauthor=true&cauthor_uid=24878536), [Kimmel PL](http://www.ncbi.nlm.nih.gov/pubmed/?term=Kimmel PL%5BAuthor%5D&cauthor=true&cauthor_uid=24878536), [Adu D](http://www.ncbi.nlm.nih.gov/pubmed/?term=Adu D%5BAuthor%5D&cauthor=true&cauthor_uid=24878536), [Ramsay M](http://www.ncbi.nlm.nih.gov/pubmed/?term=Ramsay M%5BAuthor%5D&cauthor=true&cauthor_uid=24878536), [Waddy SP](http://www.ncbi.nlm.nih.gov/pubmed/?term=Waddy SP%5BAuthor%5D&cauthor=true&cauthor_uid=24878536) et al. Understanding the rise in cardiovascular diseases in Africa: harmonising H3Africa genomic epidemiological teams and tools. Cardiovasc J Afr. 2014 May-Jun;25(3):134-6. doi: 10.5830/CVJA-2014-030. Epub 2014 May 26.

38. Ochola LI, Gitau E Challenges in Retaining Research Scientists beyond the Doctoral Level in Kenya. PLoS Neglected Trop Dis 2009; 3: e345

39. Ajuwon GA, Olorunsaye JO Knowledge, access and usage pattern of HINARI by researchers and clinicians in tertiary health institutions in south-west Nigeria. [Afr J Med Med Sci.](http://www.ncbi.nlm.nih.gov/pubmed/?term=ajuwon+knowledge+access+and+usage+pattern) 2013 Mar; 42:97-106.

40. Kebede D, Zielinski C, Mbondji PE, Sanou I, Kouvividila W, Lusamba-Dikassa P Institutional facilities in national health research systems in sub-Saharan African countries: results of structured questionnaire review. J R Soc Med 2014; DOI: 10.1177/0141076813517680

41. Koehlmoos TP, Smith R Big publishers cut access to journals in poor countries. The Lancet 2011; 377: 273 - 275

42. Nelson EA, McGuire AL The need for medical education reform: genomics and the changing nature of health information Genome Medicine. 2010; 2(3):18

43. [Mullan F](http://www.ncbi.nlm.nih.gov/pubmed/?term=Mullan F%5BAuthor%5D&cauthor=true&cauthor_uid=22778346), [Frehywot S](http://www.ncbi.nlm.nih.gov/pubmed/?term=Frehywot S%5BAuthor%5D&cauthor=true&cauthor_uid=22778346), [Omaswa F](http://www.ncbi.nlm.nih.gov/pubmed/?term=Omaswa F%5BAuthor%5D&cauthor=true&cauthor_uid=22778346), [Sewankambo N](http://www.ncbi.nlm.nih.gov/pubmed/?term=Sewankambo N%5BAuthor%5D&cauthor=true&cauthor_uid=22778346), [Talib Z](http://www.ncbi.nlm.nih.gov/pubmed/?term=Talib Z%5BAuthor%5D&cauthor=true&cauthor_uid=22778346) et al. The Medical Education Partnership Initiative: PEPFAR's effort to boost health worker education to strengthen health systems. Health Aff (Millwood). 2012 Jul;31(7):1561-72. doi: 10.1377/hlthaff.2012.0219.

44. Ojo OO, Omabe M Incorporating bioinformatics into biological science education in Nigeria: Prospects and challenges Infection, Genetics and Evolution. 2011; 11:784–787

45. [Kromberg JG](http://www.ncbi.nlm.nih.gov/pubmed/?term=Kromberg JG%5BAuthor%5D&cauthor=true&cauthor_uid=23723047), [Wessels TM](http://www.ncbi.nlm.nih.gov/pubmed/?term=Wessels TM%5BAuthor%5D&cauthor=true&cauthor_uid=23723047), [Krause A](http://www.ncbi.nlm.nih.gov/pubmed/?term=Krause A%5BAuthor%5D&cauthor=true&cauthor_uid=23723047). Roles of genetic counselors in South Africa. J Genet Couns. 2013 Dec;22(6):753-61. doi: 10.1007/s10897-013-9606-2. Epub 2013 May 31.

46. [Schoeman M](http://www.ncbi.nlm.nih.gov/pubmed/?term=Schoeman M%5BAuthor%5D&cauthor=true&cauthor_uid=23885733), [Apffelstaedt JP](http://www.ncbi.nlm.nih.gov/pubmed/?term=Apffelstaedt JP%5BAuthor%5D&cauthor=true&cauthor_uid=23885733), [Baatjes K](http://www.ncbi.nlm.nih.gov/pubmed/?term=Baatjes K%5BAuthor%5D&cauthor=true&cauthor_uid=23885733), [Urban M](http://www.ncbi.nlm.nih.gov/pubmed/?term=Urban M%5BAuthor%5D&cauthor=true&cauthor_uid=23885733). Implementation of a breast cancer genetic service in South Africa - lessons learned. S Afr Med J. 2013 Jun 25;103(8):529-33. doi: 10.7196/samj.6814.

Table 1: Frequency distribution of characteristics of publications

| **Variable** | **Frequency** | **%** |
| --- | --- | --- |
| **Country***  South Africa  Ghana  Kenya  Gambia  Nigeria  Sudan | 158  54  38  33  27  27 | 31.1  10.6  7.5  6.5  5.3  5.3 |
| **Region**  Southern Africa  East Africa  West Africa  Central Africa  2 or more regions | 182  123  151  24  30 | 35.8  24.2  29.7  4.3  5.9 |
| **Author affiliation**  First author from SSA institution  No author from SSA institution  Others (At least one author from SSA institution but not first author) | 238  45  225 | 46.9  8.9  44.2 |
| **Affiliation of first author (n=238)**  University  Research institute  Others (Ministry of Health, State Hospital, NGO) | 190  40  8 | 79.8  16.8  3.4 |
| **Disease studied****  HIV  Malaria  TB  Cancer  Cardiovascular disease | 92  103  39  31  22 | 18.1  20.3  7.7  6.1  4.3 |

*Only those countries with at least 5% proportion shown
**These five diseases were selected because of their high relative frequency in the sample and importance. Several other diseases and phenotypes constituted very small numbers and are not presented. Also, some publications focused on more than one disease

Table 2: Cross-tabulations and multivariable logistic regression of African Institution-affiliated First author and variables

|  | **Cross-tabulations** | | | **Logistic regression analysis** | |
| --- | --- | --- | --- | --- | --- |
| **Variable**** | **N** | **% AIAFA** | **P value** | **Unadjusted OR (95% CI)** | **Adjusted OR (95% CI)** |
| **Region**  Southern (ref)  East  West Africa  Central Africa  2 or more regions | 182  123  151  22  30 | 78.0  28.5  31.1  45.5  13.3 | <0.001 | 1  0.11 (0.07 – 0.19)*  0.13 (0.08 – 0.21) *  0.24 (0.10 – 0.58) *  0.04 (0.01 – 0.13) * | 1  0.12 (0.07 – 0.20)*  0.11 (0.06 – 0.19) *  0.24 (0.09 – 0.62) *  0.03 (0.01 – 0.19) * |
| **Number of countries**  1  2 or more (ref) | 468  40 | 49.1  20.0 | <0.001 | 3.87 (1.74 – 8.57)*  1 | 1.21 (0.31 – 4.73)  1 |
| **HIV-related**  Yes  No (ref) | 92  416 | 37.0  49.0 | 0.036 | 0.61 (0.38 – 0.97)*  1 | 0.30 (0.16 – 0.54)*  1 |
| **Malaria-related**  Yes  No (ref) | 103  405 | 27.2  51.9 | <0.001 | 0.35 (0.22 – 0.56)*  1 | 0.61 (0.35 – 1.07)  1 |
| **TB-related**  Yes  No | 39  469 | 51.3  46.5 | 0.564 |  |  |
| **Cancer-related**  Yes  No (ref) | 31  477 | 64.5  45.7 | 0.042 | 2.16 (1.01 – 4.61)*  1 | 1.07 (0.44 – 2.63)  1 |
| **Cardiovascular diseases**  Yes  No (ref) | 22  486 | 68.2  45.9 | 0.040 | 2.53 (1.01 – 6.31)*  1 | 1.02 (0.34 – 3.01) |
| **Year of publication** |  |  |  | 1.02 (0.96 – 1.09) | 1.04 (0.97 – 1.12) |

*Significant at 5% level of significance

**ref - Reference category for logistic regression

Identification

Literature search: Search term ‘Sub-Saharan Africa’ from the HuGE database

(n = 750)

Articles excluded (n = 116) published before Jan 2004 and after Dec 2013

Screening

Full text articles screened (n = 635)

Eligibility

Full text articles excluded (n=127)

1. Articles from Tunisia in North Africa
2. Articles including any population outside SSA
3. Publications using exclusively publicly available database such as CEPH-HGDP and HapMap
4. Publications using SSA individuals living outside SSA
5. Articles in languages other than English and French

Included

Full text review for final analysis (n = 508)

Figure 1: PRISMA (Preferred Items for Systematic Reviews and Meta-analysis) flow diagram for searching and extracting data


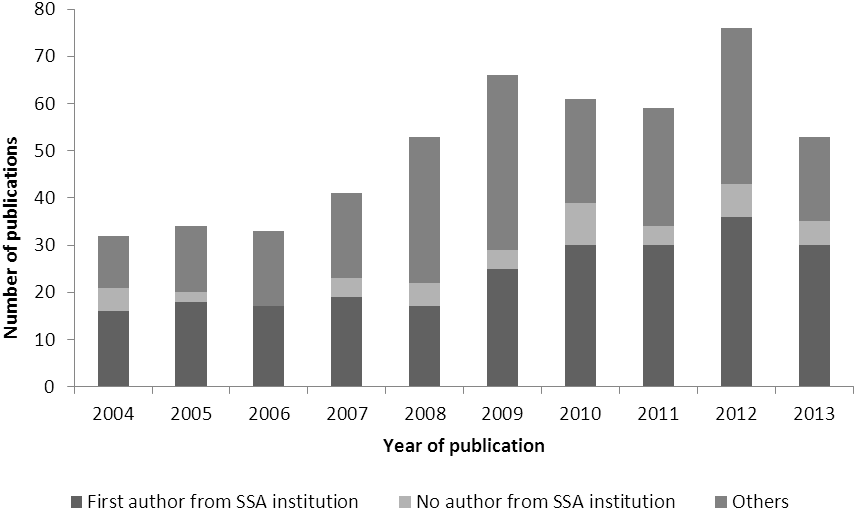


Figure 2: Trends in number of genomic epidemiology publications with author affiliated with an African institution
